# Vosoritide therapy in Brazil: Consensus on the Diagnosis, Monitoring, and Management of Achondroplasia

**DOI:** 10.1186/s13023-026-04519-8

**Published:** 2026-08-06

**Authors:** Luiz Claudio Gonçalves de Castro, Julienne Angela Ramires de Carvalho, Crésio de Aragão Dantas Alves, Têmis Maria Félix, Ana Paula Bordallo, Ricardo Fernando Arrais, Erlane Marques Ribeiro, Paula Frassinetti Vasconcelos de Medeiros, Ivana van der Linden Nader, Lena Stilianidi Garcia, Wagner Antonio da Rosa Baratela, Genoir Simoni, Guido de Paula Colares Neto, Rodrigo Ambrosio Fock, Louise Cominato, Paulo Ferrez Collett-Solberg, Kênia Maria de Souza Rio, Juliana Lopes Yamin, Juan Clinton Llerena Jr

**Affiliations:** 1 Department of Pediatrics, Faculdade de Medicina da Universidade de Brasilia (UnB), Brasilia, Brazil; 2 Pediatric Endocrinology Unit, Departament of Pediatrics, Complexo Hospital das Clínicas., Universidade Federal do Paraná (UFPR), Curitiba, Brazil; 3 Pediatric Endocrinology Unit, Hospital das Clínicas da Faculdade de Medicina, Universidade Federal da Bahia (UFBA), Salvador, Bahia Brazil; 4 Medical Genetics Service, Hospital de Clínicas de Porto Alegre, Porto Alegre, RS Brazil; 5 Pediatric Endocrinology Unit, Department of Internal Medicine, Hospital Universitário Pedro Ernesto, Universidade do Estado do Rio de Janeiro, Rio de Janeiro, Brazil; 6 Pediatric Endocrinology Unit, Department of Pediatrics, Hospital Universitário Professor Onofre Lopes, Universidade Federal do Rio Grande do Norte (UFRN/EBSERH), Natal, Brazil; 7 Medical Genetics Reference Service, Hospital Infantil Albert Sabin, Fortaleza, Ceará Brazil; 8 Academic Unit of Medicine, Hospital Universitário Alcides Carneiro, Universidade Federal de Campina Grande, Campina Grande, Paraíba, Brazil; 9 Associação de Pais e Amigos dos Excepcionais (APAE Anápolis), Anápolis, Goiás Brazil; 10 Rare Diseases Reference Service and Genetic Counseling Service, Hospital Bettina Ferro de Souza, Complexo Hospitalar Universitário da Universidade Federal do Pará, Belém, Pará Brazil; 11 Medical Genetics Department, Fleury Group, Hospital Sírio-Libanês, São Paulo, Brazil; 12 Pediatric Endocrinology Department, Hospital Infantil Joana de Gusmão, Florianópolis, Santa Catarina Brazil; 13 Instituto da Criança, Hospital das Clínicas, Faculdade de Medicina da Universidade de São Paulo (ICr-HCFMUSP), São Paulo, Brazil; 14 Center for Medical Genetics, Department of Morphology and Genetics, Universidade Federal de São Paulo (UNIFESP), São Paulo, Brazil; 15 Endocrinology Unit, Instituto da Criança, Hospital das Clínicas, Faculdade de Medicina da Universidade de São Paulo (ICr-HCFMUSP), São Paulo, Brazil; 16 Pediatric Endocrinology Division, Department of Internal Medicine, Faculdade de Ciências Médicas, Universidade do Estado do Rio de Janeiro (UERJ), Rio de Janeiro, Brazil; 17 ANNABRA – Associação de Nanismo do Brasil, Rio de Janeiro, Brazil; 18 Instituto Nacional de Nanismo, Goiânia, Goiás Brazil; 19 Centro de Genética Médica & Serviço de Referência de Doenças Raras, Instituto Nacional Fernandes Figueira, Fiocruz, Rio de Janeiro, Brazil; 20 Faculdade de Medicina de Petrópolis, UNIFASE, Rio de Janeiro, Brazil; 21 Interdisciplinary Laboratory of Biosciences - CT-34/17, Faculdade de Medicina da Universidade de Brasilia (UnB), Campus Universitario Darcy Ribeiro, DF 70910-900 Brasilia, Brazil

**Keywords:** Achondroplasia, *FGFR3*, Vosoritide, Skeletal dysplasia, Multidisciplinary care, Consensus guidelines

## Abstract

**Background:**

Achondroplasia is the most common non-lethal disproportionate skeletal dysplasia and is associated with significant lifelong medical, functional, and psychosocial complications. Advancements in the understanding of its molecular pathogenesis have facilitated the development of targeted therapies. Vosoritide, the first precision pharmacological intervention, has been shown to significantly enhance annualized growth velocity and improve body proportionality in pediatric patients with open epiphyseal plates. Currently, vosoritide is the only disease-modifying therapy approved by the Brazilian Health Regulatory Agency for the treatment of patients with achondroplasia and open growth plates. Thus, this Consensus Statement was developed to provide evidence-based, context-specific guidance for the diagnosis, monitoring, and management of individuals with achondroplasia treated with vosoritide. A multidisciplinary Guideline Development Group (GDG), comprising specialists from multiple regions of Brazil and patient representatives, formulated recommendations through a structured process that included literature review, expert discussion, and a two-round Delphi survey.

**Results:**

The final consensus presents comprehensive recommendations spanning pre-treatment evaluation, initiation criteria, dose selection, education and consent, monitoring, dose adjustment, discontinuation, and post-treatment follow-up. Key principles include confirmation of diagnosis through genetic testing, early initiation of vosoritide in eligible patients, systematic assessment of growth, complications, and quality of life, ongoing multidisciplinary care throughout life, and shared decision-making with patients and caregivers.

**Conclusions:**

This consensus aims to support uniform, high-quality clinical practice across Brazil, promote the safe and effective use of vosoritide, and improve long-term outcomes for individuals with achondroplasia.

**Clinical trial number:**

Not applicable.

**Supplementary Information:**

The online version contains supplementary material available at https://doi.org/10.1186/s13023-026-04519-8.

## Background

Achondroplasia is the most common livebirth form of disproportionate skeletal dysplasia associated with short stature, with an estimated prevalence of 1 in 10,000 to 1 in 30,000 live births, affecting approximately 360,000 individuals worldwide [1–3]. This rare autosomal dominant disorder is primarily caused by the heterozygous p.Gly380Arg pathogenic variant in the fibroblast growth factor receptor 3 (*FGFR3*) gene, which occurs in over 99% of cases [1–3]. While 20% of cases are inherited, about 80% result from *de novo* mutations, more likely linked to advanced paternal age [2, 3]. 

Primarily expressed in condensed mesenchyme, chondrocytes, osteoblasts, and osteoclasts, *FGFR3* acts within a broader signaling network as a critical regulator of skeletal formation, growth, and remodeling [1]. The *FGFR3* gain-of-function pathogenic variant causes constant activation of the RAS-MAPK signaling pathway, leading to excessive inhibition of chondrocyte proliferation and differentiation in the growth plate and, as a result, impairing endochondral ossification [1, 2]. 

Clinically, individuals present with severe short stature, primarily resulting from rhizomelic limb shortening, accompanied by macrocephaly, midfacial hypoplasia, thoracolumbar kyphosis, genu varum, and fingers arranged in a trident-like fashion, along with characteristic radiographic findings [2, 3]. Although cognitive development is typically preserved, significant medical complications may arise at various stages of life, including foramen magnum stenosis, sleep-related breathing disorders, recurrent otitis media, spinal stenosis, and orthopedic deformities. These complications can substantially affect morbidity and mortality [2, 4, 5]. In addition, a negative impact on quality of life and functionality is observed. Throughout their lives, patients experience impairments across multiple domains, including physical, social, emotional, and professional [6, 7]. Regarding functionality, anatomical limitations (e.g., short limbs and joint restrictions) may lead to hygiene-related issues, as they impair self-care activities such as dressing, bathing, and toileting [8, 9]. Brazilian clinical data highlight early manifestations, including neonatal motor delays and increased respiratory morbidity, underscoring the need for early diagnosis and specialized multidisciplinary follow‑up [2, 5, 10]. 

Historically, management relied primarily on surgery and nonspecific interventions with inconsistent outcomes. The advent of vosoritide, a C‑type natriuretic peptide analog that counteracts FGFR3 overactivity, has transformed the therapeutic landscape. Since its approval in 2021, vosoritide has demonstrated sustained benefits on annualized growth velocity, supported by randomized controlled trials and long-term extension data, together with evidence from extension studies up to five years of follow-up showing improvement in body proportionality, with a favorable safety profile [2, 3]. In this context, while international guidelines exist, the effective implementation of precision therapy for achondroplasia in Brazil faces critical barriers that require a structured national approach. The Brazilian landscape is defined by profound regional disparities in specialized infrastructure and access to diagnostic tools, which can delay the detection of life-threatening complications. This consensus, related to the use of the single therapeutic strategy available in the country, addresses this implementation gap by providing a standardized, evidence-based framework tailored to the country’s socioeconomic and logistical realities, ensuring that therapeutic innovation translates into equitable clinical benefits for all Brazilian patients [1, 2, 5]. 

This consensus was developed to provide recommendations for the diagnosis, management, and longitudinal follow-up of individuals with achondroplasia before, during, and after vosoritide treatment, the only disease-modifying therapy approved in Brazil at the time of its development.

## Methods

### Participants

Clinical specialists were selected based on predefined criteria, including documented expertise in skeletal dysplasia — particularly achondroplasia — and prior experience with vosoritide, either through clinical practice, participation in clinical trials, or management of patients receiving vosoritide therapy. The Guideline Development Group (GDG) was composed of 18 professionals from multiple Brazilian regions. The panel included clinical geneticists and pediatric endocrinologists. To incorporate patient perspectives, the GDG was supplemented by two representatives from patient association groups.

### Consensus development

A focused search of the published literature was undertaken to identify existing guidance on achondroplasia and to inform the breadth and priorities of this Consensus Statement. During a series of preliminary virtual discussions, a steering committee composed of five members of the GDG agreed on the methodological framework and overall objectives for the guideline. The steering committee then convened in virtual meetings in April 2025 to draft preliminary consensus statements informed by their clinical expertise and the perspectives of a patient representative. These draft statements were subsequently advanced to the Delphi evaluation stage.

### Delphi survey

Validation of the draft statements was carried out using a Delphi voting procedure. GDG members were asked to vote on every statement, with the option to decline participation when a topic fell outside their professional domain. Each guidance statement was evaluated using a five-point Likert scale (totally disagree, partially disagree, neither agree nor disagree, partially agree, totally agree). Consensus was defined as more than 80% of panel members selecting either “partially agree/totally agree” or “totally disagree/partially disagree”. For each final recommendation, both the level of Delphi agreement and the recommendation strength are reported. Agreement was expressed as the proportion of participating panelists who endorsed the statement (“partially agree/totally agree”). The level of agreement defined the strength of recommendation: >90% agreement was a ‘Strong recommendation’ and > 80% to ≤ 90% was a ‘Moderate recommendation’.

### Delphi outcomes

All members completed both Delphi rounds. Based on the predefined consensus criteria, all the statements achieved consensus during the first round, all of which reached positive consensus (> 80% partially agree or totally agree). However, 31 statements were resubmitted for evaluation in round 2 because the steering committee proposed substantial changes after the results of round 1. In the second round, all statements met consensus, with minor changes being performed.

### Guideline recommendations

The final version of the document sets of 55 consensus statements. It is essential to recognize that national regulatory and reimbursement systems can differ, and local regulations should take precedence when they are more comprehensive than these guidelines. A summary of the key practical actions and considerations for healthcare services and professionals regarding the implementation and oversight of vosoritide therapy is presented in Table 1. Since all the steps in the development of the recommendations were performed in Portuguese, Supplementary Table 1 shows the final set of statements in the original language. The following recommendations are the result of a rigorous, independent, and structured two-round Delphi process. To ensure clinical integrity and minimize bias, each statement required a high level of agreement (> 80%) among a multidisciplinary panel of 18 specialists and representatives from national patient associations. This collaborative and independent framework ensures that the focus remains strictly on the medical needs of Brazilian patients.

**Table 1:** Proposed recommendations, their strength, and concordance proportion

	Recommendation strength	Delphi
**Before initiating vosoritide therapy**		
***Diagnosis***		
**R1**. Confirm achondroplasia by genetic testing before prescribing vosoritide.	Strong	100%
**R2**. Patients meeting the minimum age specified in the package insert approved by the Brazilian Health Regulatory Agency (ANVISA) and presenting open epiphyseal plates should be considered eligible.	Strong	100%
**R3**. Whenever possible, treatment should be initiated early, in accordance with the locally approved prescribing information, considering the most current and robust evidence regarding the effects of the medication on craniofacial features, foramen magnum stenosis, body segment disproportionality, and the management of chronic musculoskeletal pain.	Strong	100%
**R4**. Initiating treatment in individuals with a growth velocity below 1.5 cm/year is likely to confer limited clinical benefit. In such cases, a careful discussion with the patient and family is essential to assess the residual potential for response and to determine whether there is sufficient clinical justification to initiate therapy.	Strong	87.5%
**R5**. In adolescents with closed growth plates, in adults, or in situations in which vosoritide cannot be used, patients and families should be counseled on the importance of lifelong follow-up and multidisciplinary care.	Strong	100%
**R6**. An initial in-person consultation should be conducted, preferably at a specialized center, to perform a comprehensive clinical assessment and to establish a multidisciplinary follow-up plan.	Strong	100%
**R7**. Laboratory tests and complementary assessments recommended by the national consensus should be requested, or patients should be referred accordingly for their completion.	Strong	100%
***Assessments***		
**R8**. Cranial magnetic resonance imaging, including visualization of the craniocervical junction and the foramen magnum, is recommended for patients of all ages, particularly those younger than 1 year.	Strong	100%
**R9**. Request radiographs of the hands and wrists or knee radiographs (distal femur and proximal tibia) in pubertal patients to document growth plate maturation status.	Strong	100%
***Education***		
**R10.** Updated educational materials should be provided, explaining the mechanism of action, preparation, daily administration, storage, and potential adverse events (including hypotension) associated with vosoritide.	Strong	100%
**R11**. Identify and train the individual responsible for daily administration.	Strong	100%
**R12.** Maintain an open communication channel to address questions, correct misconceptions, and update patients/caregivers regarding new evidence.	Strong	100%
***Consenting***		
**R13.** The decision to use vosoritide must be discussed and shared with all patients/caregivers.	Strong	100%
**R14.** Active participation of patients (when developmental maturity allows) and families in the therapeutic decision-making process should be encouraged, with respect for patient autonomy. Information about achondroplasia and its treatment should be communicated directly to the patient whenever possible, using age-appropriate terminology to ensure comprehension, and not solely to parents or caregivers.	Strong	100%
***Execution***		
**R15**. Support strategies should be planned and implemented to promote treatment adherence and regular attendance at follow-up visits.	Strong	100%
***Expectations***		
**R16**. Individualized goals should be defined for each patient, considering growth, motor function development, and the expected gain in annualized growth velocity according to age group.	Strong	100%
**R17**. Periodically review expectations and objectives, adjusting the care plan according to clinical evolution.	Strong	100%
***Referral***		
**R18**. Early referral to specialized centers should be pursued whenever possible, with the aim of ensuring regular follow-up and integrated access to multidisciplinary specialists.	Strong	100%
**Initiation of vosoritide therapy**		
***Indications***		
**R19**. Vosoritide should be initiated early in children with achondroplasia, in accordance with the prescribing information approved by the Brazilian Health Regulatory Agency (ANVISA), provided that epiphyseal growth plates are open, and treatment should be maintained throughout the entire growth period.	Strong	100%
**R20**. Determine the initial daily dose based on body weight and select the presentation (of the three available) that best matches the patient’s weight.	Strong	100%
***Evaluations***		
**R21.** Perform a complete clinical evaluation and record, using standardized methods, the following essential anthropometric variables before the first dose: weight, height/length, head circumference, arm span, and sitting height/crown-rump length; also, determine pubertal stage according to Tanner.	Strong	100%
**R22**. Relevant complications should be identified and documented through appropriate complementary examinations, and, whenever feasible, a validated quality-of-life assessment instrument should be applied (see Supplementary Table 2).	Strong	93.75%
***Training and instructions***		
**R23.** Provide training, preferably in person, on the administration technique of vosoritide, conducted by a qualified healthcare professional.	Strong	100%
**R24**. Patients and caregivers should be counseled on the signs and symptoms of hypotension (a possible adverse event of vosoritide), as well as on strategies for its prevention and management.	Strong	100%
**R25**. Inform about the potential adverse events of vosoritide and emphasize the importance of reporting any incidents.	Strong	100%
**R26**. Patients and families should be counseled on situations that warrant temporary interruption of treatment, and a written letter containing this information should be provided for presentation to healthcare professionals involved in care outside routine outpatient visits.	Strong	100%
**R27**. Patients and families should be advised on the feasibility and potential benefits of engaging in physical activity throughout the course of treatment.	Strong	100%
***Execution***		
**R28**. Whenever feasible, the first dose of vosoritide should be administered in a healthcare setting under the supervision of a qualified healthcare professional.	Strong	100%
**R29**. Subsequent administrations may be performed at the healthcare facility if the patient or family expresses insecurity or requires additional training time.	Strong	100%
**R30**. Home storage conditions of the medication should be verified through discussion with the patient or caregiver.	Strong	100%
**Monitoring and evaluation during vosoritide therapy**		
***Adherence and execution***		
**R31**. At every visit, confirm preparation and administration technique, verify dosing regularity, and identify supply or administration issues. If adherence is suspected to be low, investigate the cause and shorten intervals between visits.	Strong	100%
**R32**. When treatment adherence remains unsatisfactory despite targeted interventions, the frequency of follow-up should be increased, and continuation of therapy should be re-evaluated.	Strong	100%
***Follow-up***		
**R33**. Caregivers should be contacted one week after therapy initiation to reinforce practical aspects and, whenever feasible, to schedule an in-person follow-up visit to review home administration of the medication and address questions.	Strong	100%
**R34**. A follow-up visit with the prescribing physician should be conducted three months after the first dose. Thereafter, visit frequency should be adjusted according to age: • 0–2 years → every 3 months; • 3–5 years → every 4–6 months; • >5 years → every 6 months.	Strong	93.75%
**R35**. Between scheduled visits, maintain contact by phone or telemedicine as needed, and consider socioeconomic factors when establishing follow-up schedules.	Strong	100%
***Assessments***		
**R36.** At each visit, weight, height/length, body mass index (BMI), head circumference, arm span, and sitting height (or crown–rump length) should be recorded using achondroplasia-specific growth charts. Annualized growth velocity should be calculated.	Strong	100%
**R37.** Assess Tanner pubertal stage at all visits; request bone age if there are signs of puberty and repeat annually during puberty to monitor epiphyseal plate closure.	Strong	100%
**R38.** Potential complications and comorbidities should be investigated when the response to treatment is below expectations.	Strong	100%
**R39.** A validated quality-of-life assessment instrument for the Brazilian population should be applied, preferably every 12 months, including inventories to assess chronic pain.	Strong	100%
**R40.** Request repeat MRI or polysomnography only when clinically indicated.	Strong	100%
**R41.** Evaluate aspects related to the mental health of patients/families, particularly signs and symptoms of anxiety and depression, referring for specialized evaluation when necessary.	Strong	100%
***Pharmacovigilance***		
**R42.** Monitor adverse events at every visit; instruct caregivers to report serious events immediately to the responsible physician.	Strong	100%
**R43.** Review concomitant medications at each visit to identify potential interactions.	Strong	100%
***Dose adjustment***		
**R44.** Evaluate the need for vosoritide dose adjustment based on weight changes, following the package insert recommendations.	Strong	100%
**R45.** At each visit, reassess response to treatment and individualized goals regarding growth and functional gains.	Strong	100%
**Discontinuation of vosoritide therapy**		
***Criteria***		
**R46**. Discontinue vosoritide permanently in the presence of growth velocity ≤ 1.5 cm per year (as verified by repeated and validated measurements to rule out error) and/or complete epiphyseal plate closure.	Strong	100%
**R47**. Therapy may also be discontinued if the patient reaches a height previously defined as satisfactory for individual goals.	Strong	100%
**R48**. Discontinue when, after multiple assessments and review of adherence, there is no clinically meaningful gain in growth or functionality justifying continuation, or when there is unexplained functional decline/pain associated with underlying conditions.	Strong	100%
**R49**. Stop vosoritide if the patient or caregiver does not tolerate daily injections despite adequate technical and psychological support, or if they voluntarily choose to discontinue; provide counseling on therapeutic alternatives and multidisciplinary support.	Strong	100%
**R50**. Temporarily interrupt therapy in situations increasing the risk of hypotension (surgical procedures involving fluid restriction, vomiting, diarrhea, or other conditions reducing intake or increasing fluid loss).	Strong	100%
**R51.** In the event of pregnancy, treatment should be discontinued due to the lack of data supporting the safety of its use during pregnancy.	Strong	100%
**Post-vosoritide monitoring**		
***Guidance***		
**R52**. Provide guidance on the need for lifelong regular clinical follow-up upon the completion of vosoritide treatment.	Strong	100%
**R53**. Plan the transition from pediatric care to adult specialty services in a structured manner, initiating guidance during adolescence and ensuring continuity without loss to follow-up.	Strong	100%
***Assessment***		
**R54**. At each visit, frequent complications of achondroplasia should be systematically assessed within a structured, multidisciplinary approach, with referral for specialized evaluation when necessary.	Strong	100%
***Education***		
**R55**. Provide educational resources to patients/caregivers to support informed decision-making throughout adulthood, emphasizing the autosomal dominant inheritance pattern of achondroplasia and its implications for family planning within a genetic counseling context.	Strong	100%

### Before initiating vosoritide therapy

#### Diagnosis

##### R1

Confirm achondroplasia by genetic testing before prescribing vosoritide. (strong recommendation, Delphi 100%)

##### R2

Patients meeting the minimum age specified in the package insert approved by the Brazilian Health Regulatory Agency (ANVISA) and presenting open epiphyseal plates should be considered eligible. (strong recommendation, Delphi 100%)

##### R3

Whenever possible, treatment should be initiated early, in accordance with the locally approved prescribing information, considering the most current and robust evidence regarding the effects of the medication on craniofacial features, foramen magnum stenosis, body segment disproportionality, and the management of chronic musculoskeletal pain. (strong recommendation, Delphi 100%)

##### R4

Initiating treatment in individuals with a growth velocity below 1.5 cm/year is likely to confer limited clinical benefit. In such cases, a careful discussion with the patient and family is essential to assess the residual potential for response and to determine whether there is sufficient clinical justification to initiate therapy. (strong recommendation, Delphi 87.5%)

##### R5

In adolescents with closed growth plates, in adults, or in situations in which vosoritide cannot be used, patients and families should be counseled on the importance of lifelong follow-up and multidisciplinary care. (strong recommendation, Delphi 100%)

##### R6

An initial in-person consultation should be conducted, preferably at a specialized center, to perform a comprehensive clinical assessment and to establish a multidisciplinary follow-up plan. (strong recommendation, Delphi 100%)

##### R7

Laboratory tests and complementary assessments recommended by the national consensus should be requested, or patients should be referred accordingly for their completion. (strong recommendation, Delphi 100%)

Although the achondroplasia phenotype is frequently distinct, significant overlaps with other skeletal dysplasias can occur—particularly in infants or mild cases. Consequently, molecular testing is essential to ensure diagnostic accuracy, allowing clinicians to distinguish achondroplasia from phenotypic mimics and align clinical management with the achondroplasia population-specific evidence base.

Vosoritide initiation of therapy should occur only when the patient is expected to derive measurable benefit [11–13]. In achondroplasia, FGFR3 hyperactivation occurs well before birth, limiting the proliferative potential of the growth plate during the early years of life. Thus, the disease pathophysiology supports the benefit of early treatment [14]. Additionally, clinical study data indicate that, in children younger than 6 months, treatment was associated with increases in facial volume, paranasal sinus development, and foramen magnum area, further reinforcing the benefits of early intervention [15]. However, adherence to the minimum age and eligibility criteria specified in the product label is essential [11]. In patients who continue to exhibit linear growth—as those with a growth rate above 1.5 cm/year— and with open epiphyses, remaining growth potential can be assumed, supporting the likelihood of therapeutic responsiveness [11–13]. Considerations regarding the maintenance phase of treatment are detailed in the specific sections: “Monitoring and evaluation during vosoritide therapy” and “Discontinuation of vosoritide therapy”.

Given the multisystemic and lifelong impacts of achondroplasia, it is essential to adopt a multidisciplinary, proactive approach to the clinical and psychosocial care of individuals with achondroplasia throughout their lives [2]. Therefore, lifelong multidisciplinary management is essential to ensure optimal clinical outcomes and quality of life. When appropriate and aligned with the patient’s preferences, clinicians should provide counseling on orthopedic interventions, including corrective surgery for skeletal deformities or limb-lengthening procedures.

Foramen magnum stenosis is a common complication in achondroplasia, particularly in infants and younger children, who are at greater risk than older children and adults [2]. Cervical medullary compression is linked to increased mortality in infants and toddlers with achondroplasia [2, 16]. A comprehensive clinical evaluation is required to determine the need for complementary laboratory and imaging tests, which include cranial magnetic resonance imaging (MRI - with focus on the craniocervical junction), radiographs for bone age/epiphyseal status, and standardized anthropometrics (weight, height, head circumference, arm span, and sitting height). These assessments aim to: identify potential complications associated with achondroplasia—such as craniocervical junction stenosis, cervicomedullary compression, and sleep-related breathing disorders—and guide appropriate intervention when identified; exclude comorbid conditions that may impair growth and warrant targeted management; and detect cardiovascular abnormalities that may predispose the patient to hypotension, informing a more cautious approach at treatment initiation given the possibility of transient hypotension following vosoritide administration. In addition, hypertension is a recognized complication often secondary to nocturnal CO2 retention in patients with sleep-related breathing disorders [17]. This structured assessment framework also enhances detection and monitoring capabilities, particularly for telemedicine follow-up.

Although these investigations enhance diagnostic accuracy, variations in resource availability across different regions may limit full implementation. Restricted access to specialized tests in remote areas or in facilities with limited infrastructure can impede the timely identification of achondroplasia-related complications and associated conditions. In such settings, clinicians should adapt their approach to local constraints, prioritizing feasible evaluations and incorporating telemedicine-based screening and referral pathways to optimize care within the realities of the healthcare environment.

#### Assessments

##### R8

Cranial magnetic resonance imaging, including visualization of the craniocervical junction and the foramen magnum, is recommended for patients of all ages, particularly those younger than 1 year. (strong recommendation, Delphi 100%)

##### R9

Request radiographs of the hands and wrists or knee radiographs (distal femur and proximal tibia) in pubertal patients to document growth plate maturation status. (strong recommendation, Delphi 100%)

In cases where MRI identifies stenosis of the craniocervical junction or foramen magnum, referral to a neurosurgeon for further evaluation and management is strongly advised. Patients with achondroplasia may require foramen magnum decompression, which should be performed by a neurosurgical team with prior expertise in these surgeries [2]. 

Vosoritide is the first and only targeted therapy approved for use until growth plate closure, addressing the underlying pathway responsible for impaired bone growth in individuals with achondroplasia [18, 19]. A study demonstrated long-term growth-promoting effects of vosoritide in children, with treatment lasting up to 6 years [18]. However, comprehensive data on final adult height and individual height gain in large patient cohorts are still lacking. Improvements in body proportionality, particularly in the sitting height-to-height ratio, have been observed in extension studies with up to five years of follow-up, especially in children younger than 11–12 years of age [18]. Radiographic assessment to evaluate epiphyseal status should be performed judiciously during adolescence, specifically in conjunction with growth velocity analysis. Regular or pre-pubertal imaging is unnecessary; instead, radiography should be used selectively to determine the state of growth plate maturation [13]. Notably, treatment with vosoritide is indicated only in patients with open epiphyseal growth plates; therefore, complete epiphyseal plate closure constitutes a condition under which treatment must be discontinued, as per the ANVISA‑approved product label [20]. This approach serves as a critical measure of cost-effectiveness and clinical responsibility, ensuring that high-cost targeted therapy is initiated and maintained only for patients who can still derive measurable benefit.

#### Education

##### R10

Updated educational materials should be provided, explaining the mechanism of action, preparation, daily administration, storage, and potential adverse events (including hypotension) associated with vosoritide. (strong recommendation, Delphi 100%)

##### R11

Identify and train the individual responsible for daily administration. (strong recommendation, Delphi 100%)

##### R12

Maintain an open communication channel to address questions, correct misconceptions, and update patients/caregivers regarding new evidence. (strong recommendation, Delphi 100%)

Patients and caregivers should receive clear, accessible and updated information about various clinical management options for achondroplasia, which include multidisciplinary care, established orthopedic interventions (such as corrective surgery or limb lengthening), and targeted pharmacological therapy (vosoritide). Healthcare professionals and patient advocacy groups must stay informed with up-to-date, evidence-based education on vosoritide. As new data emerges, this information should be shared with all families, regardless of their opinions. It is also essential to inform patients and families about the potential benefits and risks of vosoritide, including the unknown effects of inconsistent dosing. This is particularly important for families who may face challenges obtaining vosoritide due to complex legal processes [13]. Thus, it is important that patients and their families/caregivers have access to an easy-to-read document before any decision to treat is made.

#### Consenting

##### R13

The decision to use vosoritide must be discussed and shared with all patients/caregivers. (strong recommendation, Delphi 100%)

##### R14

Active participation of patients (when developmental maturity allows) and families in the therapeutic decision-making process should be encouraged, with respect for patient autonomy. Information about achondroplasia and its treatment should be communicated directly to the patient whenever possible, using age-appropriate terminology to ensure comprehension, and not solely to parents or caregivers. (strong recommendation, Delphi 100%)

Shared decision-making in pediatric care involves determining if multiple medically reasonable options are available, assessing which option offers the most benefit to the patient while considering the family’s impact, and understanding the family’s values and preferences. Peer support and family organizations can also support or inform decision-making [21]. 

#### Planning

##### R15

Support strategies should be planned and implemented to promote treatment adherence and regular attendance at follow-up visits. (strong recommendation, Delphi 100%)

##### R16

Individualized goals should be defined for each patient, considering growth, motor function development, and the expected gain in annualized growth velocity according to age group. (strong recommendation, Delphi 100%)

##### R17

Periodically review expectations and objectives, adjusting the care plan according to clinical evolution. (strong recommendation, Delphi 100%)

##### R18

Early referral to specialized centers should be pursued whenever possible, with the aim of ensuring regular follow-up and integrated access to multidisciplinary specialists. (strong recommendation, Delphi 100%)

Patients with achondroplasia and their parents/caregivers typically have two primary objectives for pharmacological treatment: alleviating complications associated with the condition and increasing height to enhance functional abilities and psychosocial well-being. Key concerns include chronic pain, spinal surgeries, ear, nose, and throat issues, and neurological complications. Achieving greater height may foster independence, improve social integration, and reduce stigma. However, decisions are also influenced by the significance of height to personal identity and concerns that treatment may not normalize all clinical aspects or complications of the condition [22]. 

### Initiation of vosoritide therapy

#### Indications

##### R19

Vosoritide should be initiated early in children with achondroplasia, in accordance with the prescribing information approved by the Brazilian Health Regulatory Agency (ANVISA), provided that epiphyseal growth plates are open, and treatment should be maintained throughout the entire growth period. (strong recommendation, Delphi 100%)

##### R20

Determine the initial daily dose based on body weight and select the presentation (of the three available) that best matches the patient’s weight. (strong recommendation, Delphi 100%)

An open-label extension study evaluated the long-term efficacy and safety of daily subcutaneous vosoritide (15 µg/kg) in 119 children aged 5 years and older following the pivotal Phase 3 trial. Over six years of treatment, participants demonstrated sustained growth acceleration. Notably, improvements in body proportions were observed primarily in the younger cohort (typically those aged 11–12 years). The safety profile remained consistent with earlier findings, with no new treatment-related concerns identified [19]. 

#### Evaluations

##### R21

Perform a complete clinical evaluation and record, using standardized methods, the following essential anthropometric variables before the first dose: weight, height/length, head circumference, arm span, and sitting height/crown-rump length; also, determine pubertal stage according to Tanner. (strong recommendation, Delphi 100%)

##### R22

Relevant complications should be identified and documented through appropriate complementary examinations, and, whenever feasible, a validated quality-of-life assessment instrument should be applied (see Supplementary Table 2). (strong recommendation, Delphi 93.75%)

Several efforts have been made to develop specific charts to evaluate individuals with achondroplasia worldwide [23, 24]. Thus, the use of achondroplasia-specific growth charts — such as those providing normative data for body proportions and BMI available at www.achondroplasia-growthcharts.com — is essential to ensure clinical precision during assessment [25].

Individuals with achondroplasia experience a significantly lower health-related quality of life (HRQoL) than the general population. This disparity is driven by severe and disproportionate short stature and rhizomelic shortening of the upper limbs, hindering essential daily activities and reducing independence from childhood through adulthood. A final height of less than 140 cm is a critical threshold that likely significantly impacts physical HRQoL. Beyond these functional limitations, HRQoL may be compromised by a heavy surgical burden (including ear, nose, and throat and orthopedic procedures), chronic pain, restricted employment opportunities often stemming from spinal stenosis, and, in some cases, decreased life expectancy [16]. The Lifetime Impact Study for Achondroplasia (LISA) assessed HRQoL among patients with achondroplasia in Argentina, Brazil and Colombia using Quality of Life in Short Stature Youth (QoLISSY) and Pediatric Quality of Life Inventory (PedsQL) questionnaires revealed lower scores once compared with average stature children [26]. Although only PedsQL is formally validated to the Brazilian context, both instruments are used and can be considered in the patient’s clinical follow-up [26, 27]. Currently, there is no achondroplasia-specific quality of life instrument formally validated for the Brazilian population.

#### Training and instructions

##### R23

Provide training, preferably in person, on the administration technique of vosoritide, conducted by a qualified healthcare professional. (strong recommendation, Delphi 100%)

##### R24

Patients and caregivers should be counseled on the signs and symptoms of hypotension (a possible adverse event of vosoritide), as well as on strategies for its prevention and management. (strong recommendation, Delphi 100%)

##### R25

Inform about the potential adverse events of vosoritide and emphasize the importance of reporting any incidents. (strong recommendation, Delphi 100%)

##### R26

Patients and families should be counseled on situations that warrant temporary interruption of treatment, and a written letter containing this information should be provided for presentation to healthcare professionals involved in care outside routine outpatient visits. (strong recommendation, Delphi 100%)

##### R27

Patients and families should be advised on the feasibility and potential benefits of engaging in physical activity throughout the course of treatment. (strong recommendation, Delphi 100%)

##### R28

Whenever feasible, the first dose of vosoritide should be administered in a healthcare setting under the supervision of a qualified healthcare professional. (strong recommendation, Delphi 100%)

##### R29

Subsequent drug administrations may be performed at the healthcare facility if the patient or family expresses insecurity or requires additional training time. (strong recommendation, Delphi 100%)

##### R30

Home storage conditions of the medication should be verified through discussion with the patient or caregiver. (strong recommendation, Delphi 100%)

The visit discussing administration technique of vosoritide should cover injection technique, tolerance, site management, and adverse effects. The consultation should also address supply management, including pharmacy coordination and home storage. During follow-up visits, focus on the patient’s well-being, emotional health, adherence, and daily challenges related to injections [13]. 

### Monitoring and evaluation during vosoritide therapy

#### Monitoring

##### R31

At every visit, confirm preparation and administration technique, verify dosing regularity, and identify supply or administration issues. If adherence is suspected to be low, investigate the cause and shorten intervals between visits. (strong recommendation, Delphi 100%)

##### R32

When treatment adherence remains unsatisfactory despite targeted interventions, the frequency of follow-up should be increased, and continuation of therapy should be re-evaluated. (strong recommendation, Delphi 100%)

##### R33

Caregivers should be contacted one week after therapy initiation to reinforce practical aspects and, whenever feasible, to schedule an in-person follow-up visit to review home administration of the medication and address questions. (strong recommendation, Delphi 100%)

##### R34

A follow-up visit with the prescribing physician should be conducted three months after the first dose. Thereafter, visit frequency should be adjusted according to age: • 0–2 years → every 3 months; • 3–5 years → every 4–6 months; • >5 years → every 6 months. (strong recommendation, Delphi 93.75%)

##### R35

Between scheduled visits, maintain contact by phone or telemedicine as needed, and consider socioeconomic factors when establishing follow-up schedules. (strong recommendation, Delphi 100%)

Medical complications related to achondroplasia can occur at any stage of life and require continuous monitoring and management. Individuals with achondroplasia must receive ongoing follow-up care from a multidisciplinary team, guided by a healthcare professional with specialized knowledge of the condition. Regular evaluations are critical during the first two years of life, when early intervention and close monitoring help address potential issues quickly and improve long-term health outcomes. This approach ensures that the unique needs of individuals with achondroplasia are met throughout their lifespan [2]. 

#### Follow-up

##### R36

At each visit, weight, height/length, body mass index (BMI), head circumference, arm span, and sitting height (or crown–rump length) should be recorded using achondroplasia-specific growth charts. Annualized growth velocity should be calculated. (strong recommendation, Delphi 100%)

##### R37

Assess Tanner pubertal stage at all visits; request bone age if there are signs of puberty and repeat annually during puberty to monitor epiphyseal plate closure. (strong recommendation, Delphi 100%)

##### R38

Potential complications and comorbidities should be investigated when the response to treatment is below expectations. (moderate recommendation, Delphi 100%)

##### R39

A validated quality-of-life assessment instrument for the Brazilian population should be applied, preferably every 12 months, including inventories to assess chronic pain. (strong recommendation, Delphi 100%)

##### R40

Request repeat MRI or polysomnography only when clinically indicated. (strong recommendation, Delphi 100%)

##### R41

Evaluate aspects related to the mental health of patients/families, particularly signs and symptoms of anxiety and depression, referring for specialized evaluation when necessary. (strong recommendation, Delphi 100%)

Routine monitoring of outcomes, as part of standard care, should persist as recommended, including assessments for foramen magnum stenosis, sleep-disordered breathing, spinal issues, leg deformities, hearing, developmental milestones, and functional performance. The frequency and method of ongoing follow-up should be tailored to the patient’s age and condition. More frequent reviews may be necessary to ensure continued adherence to treatment and to assess the patient and family’s commitment to ongoing therapy, particularly if non-compliance is suspected. Furthermore, evaluations for new comorbidities should be conducted [13]. Radiographic evaluation of skeletal maturation should be performed judiciously during puberty, and its indication must be strictly guided by the patient’s annualized growth velocity data. As previously stated in this document, the use of achondroplasia-specific growth charts is essential throughout the whole follow-up [25]. 

As treatment with vosoritide is indicated only in patients with open epiphyseal growth plates, according to the ANVISA‑approved product label, confirmation of complete epiphyseal plate closure establishes the loss of the approved indication and therefore supports treatment discontinuation [20]. This assessment provides the biological rationale required to guide discontinuation decisions and reflects appropriate clinical stewardship, ensuring that the use of high‑cost precision therapy remains aligned with regulatory conditions of use and responsible allocation of healthcare resources.

#### Pharmacovigilance

##### R42

Monitor adverse events at every visit; instruct caregivers to report serious events immediately to the responsible physician. (strong recommendation, Delphi 100%)

##### R43

Review concomitant medications at each visit to identify potential interactions. (strong recommendation, Delphi 100%)

Vosoritide is a new medication and, although research has indicated acceptable efficacy and safety, even when indicated and used correctly, unpredictable or unknown adverse events may occur. In this case, report adverse events through the VigiMed System, available on the ANVISA Portal [11]. 

#### Dose adjustment

##### R44

Evaluate the need for vosoritide dose adjustment based on weight changes, following the package insert recommendations. (strong recommendation, Delphi 100%)

##### R45

At each visit, reassess response to treatment and individualized goals regarding growth and functional gains. (strong recommendation, Delphi 100%)

Apparent clearance and volume of distribution of vosoritide increase with body weight in patients with achondroplasia (9 to 74.5 kg). The recommended dosage considers this variation and suggests using doses above (for patients between 10 and 16 kg) or below (for those over 44 kg) the “standard daily dose” of 15 mcg/kg to achieve a similar level of exposure across all weight ranges [11]. Patients should undergo regular clinical assessment every 3–6 months, tailored to their age and specific needs. These visits aim to monitor weight, growth velocity, and physical development, alongside the comprehensive global monitoring previously described. The dose should be adjusted according to the patient’s body weight [11]. 

### Discontinuation of vosoritide therapy

#### Criteria

##### R46

Discontinue vosoritide permanently in the presence of growth velocity ≤ 1.5 cm per year (as verified by repeated and validated measurements to rule out error) and/or complete epiphyseal plate closure. (strong recommendation, Delphi 100%)

##### R47

Therapy may also be discontinued if the patient reaches a height previously defined as satisfactory for individual goals. (strong recommendation, Delphi 100%)

##### R48

Discontinue when, after multiple assessments and review of adherence, there is no clinically meaningful gain in growth or functionality justifying continuation, or when there is unexplained functional decline/pain associated with underlying conditions. (strong recommendation, Delphi 100%)

##### R49

Stop vosoritide if the patient or caregiver does not tolerate daily injections despite adequate technical and psychological support, or if they voluntarily choose to discontinue; provide counseling on therapeutic alternatives and multidisciplinary support. (strong recommendation, Delphi 100%)

##### R50

Temporarily interrupt therapy in situations increasing the risk of hypotension (surgical procedures involving fluid restriction, vomiting, diarrhea, or other conditions reducing intake or increasing fluid loss). (strong recommendation, Delphi 100%)

The decision to discontinue vosoritide treatment can be made at any point after thorough counseling and discussions with both the patient and caregiver regarding treatment goals. For example, treatment may be stopped if the patient, such as a child, no longer consents or wishes to discontinue. If treatment is stopped once a patient reaches a self-defined satisfactory height (individualized goal), they must be informed that the maximum potential biological gain from the therapy may not have been fully realized. Discontinuation might also be considered if the patient shows no response to the therapy or experiences unexplained pain. When deciding to stop due to poor tolerance of injections, this should only occur after careful discussion and sufficient support for managing the injection process. It is important to highlight that the discontinuation threshold of growth velocity ≤ 1.5 cm/year is typically indicative of the postpubertal phase or growth plate maturation. Currently, no data is available on the effects of treatment interruption [13]. 

### Post-vosoritide monitoring

#### Guidance

##### R51

In the event of pregnancy, treatment should be discontinued due to the lack of data supporting the safety of its use during pregnancy. (strong recommendation, Delphi 100%)

##### R52

Provide guidance on the need for lifelong regular clinical follow-up upon the completion of vosoritide treatment. (strong recommendation, Delphi 100%)

The cessation of pharmacological treatment does not eliminate the need for multidisciplinary monitoring. Ongoing assessments should focus on the surveillance of late-onset complications Therefore, raising awareness and effectively managing these issues is crucial for promoting optimal outcomes and improving the quality of life for individuals with this condition and their families [2]. 

#### Assessment

##### R53

Plan the transition from pediatric care to adult specialty services in a structured manner, initiating guidance during adolescence and ensuring continuity without loss to follow-up. (strong recommendation, Delphi 100%)

##### R54

At each visit, frequent complications of achondroplasia should be systematically assessed within a structured, multidisciplinary approach, with referral for specialized evaluation when necessary. (strong recommendation, Delphi 100%)

##### R55

Provide educational resources to patients/caregivers to support informed decision-making throughout adulthood, emphasizing the autosomal dominant inheritance pattern of achondroplasia and its implications for family planning within a genetic counseling context. (strong recommendation, Delphi 100%)

Lifelong regular clinical follow-up remains indispensable. Throughout the clinical course of achondroplasia, patients may experience complications affecting the neurological, orthopedic, cardiovascular, otolaryngological, respiratory, and psychosocial domains. Referral to appropriate specialists for ongoing management is essential to address these comorbidities and optimize patient outcomes [2]. 

Understanding genetics and psychology can help patients and their families cope with medical information, including disease progression and treatment options. It aids in assessing hereditary patterns and exploring ways to manage the risk of recurrence within the family. Additionally, it provides accurate, unbiased information to support individuals in making informed decisions about family planning, while respecting their autonomy and considering ethical, cultural, and socioeconomic factors [12]. 

## Conclusion

This Brazilian Consensus on the Diagnosis, Monitoring, and Management of Achondroplasia with Vosoritide Therapy offers clear, evidence-based, and context-appropriate recommendations, with a particular focus on the safe and effective use of vosoritide. By integrating current scientific evidence with expert opinion and a structured consensus process, this guideline aims to promote standardized, high-quality care nationwide and ensure comprehensive, multidisciplinary, and patient‑centered management throughout the lifespan. Early detection, timely treatment, regular monitoring, and shared decision-making are essential components of optimal care. This consensus provides guidance specifically on vosoritide therapy, which remains the only disease-specific pharmacological treatment approved by ANVISA for achondroplasia in Brazil. The global therapeutic landscape is evolving rapidly, with additional agents having reached regulatory approval or advanced clinical development elsewhere. As new therapies obtain ANVISA approval, these recommendations should be reviewed to incorporate the available options and the respective benefits and limitations of each, in line with Good Clinical Practice.

## Supplementary Information

Below is the link to the electronic supplementary material.

Supplementary Material 1

Supplementary Material 2

## Data Availability

The authors confirm that all data generated or analysed during this study are included in this published article.

## Abbreviations

ANVISA: Brazilian Health Regulatory Agency
BIA: Bioelectrical Impedance Analysis
BMI: Body Mass Index
cm: Centimeter(s)
FFM: Fat-Free Mass
FGFR3: Fibroblast Growth Factor Receptor 3
GDG: Guideline Development Group
HRQoL: Health-Related Quality of Life
kg: Kilogram(s)
LISA: Lifetime Impact Study for Achondroplasia (LISA)
mcg/kg: Microgram(s) per kilogram
µg/kg: Microgram(s) per kilogram
MAPK: Mitogen-Activated Protein Kinase
MRI: Magnetic Resonance Imaging
PedsQL: Pediatric Quality of Life Inventory
QoLISSY: Quality of Life in Short Stature Youth
Ras/MAPK: Mitogen-Activated Protein Kinase
REE: Resting Energy Expenditure

## References

[1] Chen H, Zhang R, Jin M, Yang J, Chen L, Xie Y. Advances in the mechanism and therapies of achondroplasia. Genes Dis. 2025;12:101436. 10.1016/j.gendis.2024.101436. PMC12008630 40256430

[2] Savarirayan R, Ireland P, Irving M, Thompson D, Alves I, Baratela WAR, et al. International Consensus Statement on the diagnosis, multidisciplinary management and lifelong care of individuals with achondroplasia. Nat Rev Endocrinol. 2022;18:173–89. 10.1038/s41574-021-00595-x. 34837063

[3] Merchant N, Hoover-Fong J, Carroll RS. Approach to the patient with achondroplasia-new considerations for diagnosis, management, and treatment. J Clin Endocrinol Metab. 2025;110:e2309–16. 10.1210/clinem/dgaf017. PMC12187453 39813116

[4] Foreman PK, van Kessel F, van Hoorn R, van den Bosch J, Shediac R, Landis S. Birth prevalence of achondroplasia: A systematic literature review and meta-analysis. Am J Med Genet A. 2020;182:2297–316. 10.1002/ajmg.a.61787. PMC7540685 32803853

[5] Guillen-Navarro E, AlSayed M, Alves I, Ben-Omran T, Boero S, Cormier-Daire V, et al. Recommendations for management of infants and young children with achondroplasia: Does clinical practice align? Orphanet J Rare Dis. 2025;20:114. 10.1186/s13023-025-03621-7. PMC11895228 40065464

[6] Witt S, Kolb B, Bloemeke J, Mohnike K, Bullinger M, Quitmann J. Quality of life of children with achondroplasia and their parents - a German cross-sectional study. Orphanet J Rare Dis. 2019;14:194. 10.1186/s13023-019-1171-9. PMC6688231 31399110

[7] Johansen H, Andresen I-L, Naess EE, Hagen KB. Health status of adults with Short Stature: A comparison with the normal population and one well-known chronic disease (Rheumatoid Arthritis). Orphanet J Rare Dis. 2007;2:10. 10.1186/1750-1172-2-10. PMC1838413 17324297

[8] Ireland PJ, McGill J, Zankl A, Ware RS. Functional performance in young Australian children with achondroplasia. Dev Med Child Neurol. 2011;53:944–50. 10.1111/j.1469-8749.2011.04050.x. 21838822

[9] Alade Y, Tunkel D, Schulze K, McGready J, Jallo G, Ain M, et al. Cross-sectional assessment of pain and physical function in skeletal dysplasia patients. Clin Genet. 2013;84:237–43. 10.1111/cge.12045. 23106480

[10] Maia Filho JM, Medeiros L, de Freitas O, de Bringel APA, Rodrigues AC, Lima Filho JMM, de O MR, et al. Aspectos clínicos e epidemiológicos da Acondroplasia: uma série de casos do Nordeste brasileiro. Res Soc Dev. 2022;11:e12811628727. 10.33448/rsd-v11i6.28727.

[11] Ministério da Sáude (BR). Agência Nacional de Vigilância Sanitária (Anvisa). Voxzogo^®^ Product Label [Internet]. [cited 2025 Nov 19]. https://consultas.anvisa.gov.br/#/bulario/q/?nomeProduto=VOXZOGO. Accessed 19 Nov 2025.

[12] Crésio A, Salmo R, Abelardo P, Rocha BA, Azevedo AI. Acondroplasia: etiologia, apresentação clínica e tratamento (Diretriz). Rio de Janeiro, RJ; 2023.

[13] Savarirayan R, Hoover-Fong J, Ozono K, Backeljauw P, Cormier-Daire V, DeAndrade K, et al. International consensus guidelines on the implementation and monitoring of vosoritide therapy in individuals with achondroplasia. Nat Rev Endocrinol. 2025;21:314–24. 10.1038/s41574-024-01074-9. 39757323

[14] Pauli RM. Achondroplasia: a comprehensive clinical review. Orphanet J Rare Dis. 2019;14:1. 10.1186/s13023-018-0972-6. PMC6318916 30606190

[15] Savarirayan R, Wilcox WW, Harmatz P III, Polgreen JP, Tofts LE. LBMON196 A Randomized Controlled Trial Of Vosoritide In Infants And Toddlers With Achondroplasia. J Endocr Soc. 2022;6:A591–2. 10.1210/jendso/bvac150.1225.

[16] Savarirayan R, Baratela W, Butt T, Cormier-Daire V, Irving M, Miller BS, et al. Literature review and expert opinion on the impact of achondroplasia on medical complications and health-related quality of life and expectations for long-term impact of vosoritide: a modified Delphi study. Orphanet J Rare Dis. 2022;17:224. 10.1186/s13023-022-02372-z. PMC9195406 35698202

[17] Fauroux B, AlSayed M, Ben-Omran T, Boero S, Boon M, Cormier-Daire V, et al. Management of sleep-disordered breathing in achondroplasia: guiding principles of the European Achondroplasia Forum. Orphanet J Rare Dis. 2025;20:233. 10.1186/s13023-025-03717-0. PMC12083049 40375310

[18] Savarirayan R, Tofts L, Irving M, Wilcox W, Bacino CA, Hoover-Fong J, et al. Once-daily, subcutaneous vosoritide therapy in children with achondroplasia: a randomised, double-blind, phase 3, placebo-controlled, multicentre trial. Lancet. 2020;396:684–92. 10.1016/S0140-6736(20)31541-5. 32891212

[19] Savarirayan R, Irving M, Wilcox WR, Bacino CA, Hoover-Fong JE, Harmatz P, et al. Sustained growth-promoting effects of vosoritide in children with achondroplasia from an ongoing phase 3 extension study. Med (N Y). 2025;6:100566. 10.1016/j.medj.2024.11.019. 39740666

[20] BioMarin Brasil. Farmacêutica Ltda. Voxzogo ^®^ vosoritida. 2026.

[21] American Academy of Pediatrics. Shared Decision Making [Internet]. 2025. https://www.aap.org/en/practice-management/providing-patient--and-family-centered-care/shared-decision-making/.

[22] McGraw SA, Henne JC, Nutter J, Larkin AA, Chen E. Treatment Goals for Achondroplasia: A Qualitative Study with Parents and Adults. Adv Ther. 2022;39:3378–91. 10.1007/s12325-022-02190-6. PMC9239927 35672555

[23] Merker A, Neumeyer L, Hertel NT, Grigelioniene G, Mäkitie O, Mohnike K, et al. Growth in achondroplasia: Development of height, weight, head circumference, and body mass index in a European cohort. Am J Med Genet A. 2018;176:1723–34. 10.1002/ajmg.a.38853. 30070757

[24] Hoover-Fong JE, Schulze KJ, Alade AY, Bober MB, Gough E, Hashmi SS, et al. Growth in achondroplasia including stature, weight, weight-for-height and head circumference from CLARITY: achondroplasia natural history study—a multi-center retrospective cohort study of achondroplasia in the US. Orphanet J Rare Dis. 2021;16:522. 10.1186/s13023-021-02141-4. PMC8697459 34949201

[25] Neumeyer L, Merker A, Hagenäs L. Achondroplasia Growth Charts [Internet]. [cited 2026 Mar 28]. https://www.achondroplasia-growthcharts.com/. Accessed 28 Mar 2026.

[26] Llerena J, Rosselli P, Aragão A, Valenzuela C, Bertola D, Mendez Y, et al. Lifetime Impact Study for Achondroplasia (LISA): Findings from an observational and multinational study focused on health-related quality of life in individuals with achondroplasia in Latin America. Genet Med Open. 2024;2:100843. 10.1016/j.gimo.2023.100843. PMC11613863 39669637

[27] Klatchoian DA, Len CA, Terreri MTRA, Silva M, Itamoto C, Ciconelli RM, et al. Qualidade de vida de crianças e adolescentes de São Paulo: confiabilidade e validade da versão brasileira do questionário genérico Pediatric Quality of Life InventoryTM versão 4.0. J Pediatr (Rio J). 2008;84. 10.1590/S0021-75572008000400005.

