# Supplementary material for: Vosoritide therapy in Brazil: Consensus on the Diagnosis, Monitoring, and Management of Achondroplasia
Source: Orphanet J Rare Dis. 2026 Aug 6;21:396. doi: 10.1186/s13023-026-04519-8 (PMC13732916; doi:10.1186/s13023-026-04519-8)
Supplement: Supplementary file 1 [file 13023_2026_4519_MOESM1_ESM.docx]

**SUPPLEMENTARY MATERIAL**

Supplementary Table 1. Statements as developed in Brazilian Portuguese.

|  | **Recommendation strength** | **Delphi** |
| --- | --- | --- |
| **Before initiating vosoritide therapy** |  |  |
| ***Diagnosis*** |  |  |
| **R1**. Confirmar a acondroplasia por teste genético, antes de prescrever vosoritida. | **Strong** | **100%** |
| **R2**. Devem ser considerados elegíveis os pacientes que atendam à idade mínima estabelecida na bula aprovada pela Agência Nacional de Vigilância Sanitária (ANVISA) e que apresentem placas epifisárias abertas. | **Strong** | **100%** |
| **R3**. Sempre que possível, o tratamento deve ser iniciado de maneira precoce, conforme as recomendações da bula vigente no país, levando-se em consideração as evidências mais atuais e de melhor consistência disponíveis sobre o impacto da medicação nas alterações craniofaciais, na estenose do forame magno, na desproporcionalidade entre os segmentos corporais e no controle das dores musculoesqueléticas crônicas. | **Strong** | **100%** |
| **R4**. A indicação de início de tratamento para indivíduos com velocidade de crescimento inferior a 1,5 cm/ano tende a oferecer benefício limitado. Nesses casos, é essencial discutir cuidadosamente com a família e com o paciente o potencial residual de resposta, avaliando-se se há justificativa clínica para iniciar terapia. | **Strong** | **87.5%** |
| **R5**. Em adolescentes com placas de crescimento fechadas, adultos ou em situações que não é possível usar vosoritida, deve-se orientar sobre a importância do acompanhamento e do suporte multidisciplinar ao longo da vida. | **Strong** | **100%** |
| **R6**. Realizar uma consulta presencial inicial, preferencialmente em um centro especializado, para avaliação clínica completa e organização do plano de acompanhamento multidisciplinar. | **Strong** | **100%** |
| **R7**. Solicitar ou encaminhar para a realização dos exames laboratoriais e avaliações complementares recomendados pelo consenso nacional. | **Strong** | **100%** |
| ***Assessments*** |  |  |
| **R8**. Recomenda-se solicitar ressonância magnética de crânio com visualização da junção crânio cervical e do forame magno para todas as idades, especialmente em pacientes < 1 ano. | **Strong** | **100%** |
| **R9**. Solicitar radiografias de mãos e punhos ou radiografia de joelhos (fêmur distal e tíbia proximal) em pacientes púberes para documentar o estado de maturação da placa de crescimento. | **Strong** | **100%** |
| ***Education*** |  |  |
| **R10.** Disponibilizar materiais educativos atualizados, explicando mecanismo de ação, preparo, administração diária, armazenamento e potenciais eventos adversos (incluindo hipotensão) da vosoritida. | **Strong** | **100%** |
| **R11**. Identificar e treinar o responsável pela aplicação diária. | **Strong** | **100%** |
| **R12.** Manter canal aberto para responder dúvidas, corrigir informações equivocadas e atualizar pacientes/famílias sempre que surgirem novas evidências. | **Strong** | **100%** |
| ***Consenting*** |  |  |
| **R13.** A opção pela vosoritida deve ser discutida com todos os pacientes/famílias. | **Strong** | **100%** |
| **R14.** Promover a participação ativa dos pacientes/famílias no processo de tomada de decisão terapêutica, respeitando sua autonomia. As informações sobre a acondroplasia e o tratamento devem ser apresentadas diretamente ao paciente, sempre que possível, e não apenas aos pais. | **Strong** | **100%** |
| ***Execution*** |  |  |
| **R15**. Planejar estratégias de apoio que favoreçam a adesão terapêutica e assiduidade às consultas. | **Strong** | **100%** |
| ***Expectations*** |  |  |
| **R16**. Definir metas individualizadas para cada paciente, considerando crescimento, desenvolvimento das funções motoras e o ganho esperado na velocidade de crescimento anualizada, de acordo com a faixa etária. | **Strong** | **100%** |
| **R17**. Revisar expectativas e objetivos periodicamente, ajustando o plano de cuidado conforme evolução clínica. | **Strong** | **100%** |
| ***Referral*** |  |  |
| **R18**. Encaminhar, preferencialmente de forma precoce, para centros especializados, buscando acompanhamento regular e acesso integrado a especialistas. | **Strong** | **100%** |
| **Initiation of vosoritide therapy** |  |  |
| ***Indications*** |  |  |
| **R19**. Iniciar a vosoritida de forma precoce em crianças com acondroplasia, conforme a bula aprovada pela Agência Nacional de Vigilância Sanitária (ANVISA), desde que as placas epifisárias estejam abertas, mantendo-se o tratamento durante todo o período de crescimento. | **Strong** | **100%** |
| **R20**. Determinar a dose diária inicial de acordo com o peso corporal e selecionar, entre as três apresentações disponíveis, a que melhor se adeque ao peso do paciente. | **Strong** | **100%** |
| ***Evaluations*** |  |  |
| **R21.** Realizar avaliação clínica completa e medir de maneira padronizada, antes da primeira dose, as seguintes variáveis antropométricas indispensáveis: peso, estatura/comprimento, perímetro cefálico, envergadura e altura sentada/medida cabeça-nádega; além de classificar o estágio puberal segundo Tanner. | **Strong** | **100%** |
| **R22**. Identificar e documentar complicações relevantes por exames complementares e aplicar, se possível, instrumento validado de qualidade de vida, vide tabela suplementar. | **Strong** | **100%** |
| ***Training and instructions*** |  |  |
| **R23.** Prover treinamento, preferencialmente de forma presencial, sobre a técnica de administração, conduzido por profissional de saúde habilitado. | **Strong** | **100%** |
| **R24**. Orientar sobre sinais e sintomas de hipotensão, assim como estratégias de prevenção e manejo. | **Strong** | **100%** |
| **R25**. Informar os potenciais eventos adversos e enfatizar a importância de relatar qualquer incidente. | **Strong** | **100%** |
| **R26**. Orientar os pacientes/famílias sobre situações que indiquem a interrupção temporária do tratamento e fornecer carta com essas informações, para que possa ser apresentada ao médico que os atender em situações fora das consultas ambulatoriais de rotina. | **Strong** | **100%** |
| **R27**. Orientar quanto à possibilidade e os benefícios da realização de atividade física ao longo do tratamento. | **Strong** | **100%** |
| ***Execution*** |  |  |
| **R28**. Administrar a primeira dose, sempre que possível, em serviço de saúde e sob a supervisão de profissional habilitado. | **Strong** | **100%** |
| **R29**. Aplicações subsequentes podem ser realizadas na unidade de saúde caso o paciente/família manifeste insegurança ou necessidade de maior tempo de treinamento. | **Strong** | **100%** |
| **R30**. As condições de armazenamento do medicamento no domicílio devem ser verificadas em conversa com o paciente/familiar. | **Strong** | **100%** |
| **Monitoring and evaluation during vosoritide therapy** |  |  |
| ***Adherence and execution*** |  |  |
| **R31**.Em todas as consultas, confirmar a técnica de preparo e administração, verificar a regularidade da aplicação das doses e identificar eventuais falhas de abastecimento ou de aplicação. Se houver suspeita de baixa adesão, investigar a causa e reduzir o intervalo entre as consultas. | **Strong** | **100%** |
| **R32**. Quando a adesão permanecer insatisfatória, apesar das intervenções, deve-se aumentar a frequência do acompanhamento e reavaliar a continuidade do tratamento. | **Strong** | **100%** |
| ***Follow-up*** |  |  |
| **R33**. Entrar em contato com os responsáveis após uma semana do início da terapia para reforçar aspectos práticos e, se possível, agendar uma consulta presencial de retorno para revisar o manejo domiciliar da medicação e esclarecer dúvidas. | **Strong** | **100%** |
| **R34**. Realizar consulta de acompanhamento com o médico responsável pela prescrição da vosoritida três meses após a primeira dose e, posteriormente, ajustar a periodicidade das consultas conforme a idade: • 0–2 anos → a cada 3 meses; • 3–5 anos → a cada 4–6 meses; • > 5 anos → a cada 6 meses. | **Strong** | **93.75%** |
| **R35**. Entre as consultas programadas, manter contato telefônico ou teleconsulta sempre que necessário e considerar fatores socioeconômicos na definição do cronograma de consultas. | **Strong** | **100%** |
| ***Assessments*** |  |  |
| **R36.** Registrar, em cada visita, peso, estatura/comprimento, IMC, perímetro cefálico, envergadura e altura sentada/medida cabeça-nádega, utilizando curvas específicas para acondroplasia. Calcular velocidade de crescimento anualizada. | **Strong** | **100%** |
| **R37.** b. Avaliar estágio puberal de Tanner em todas as consultas; solicitar idade óssea se houver sinais de puberdade e repetir anualmente durante a puberdade para monitorar fechamento das placas epifisárias. | **Strong** | **100%** |
| **R38.** Investigar possíveis complicações e comorbidades quando a resposta ao tratamento for inferior ao esperado. | **Strong** | **100%** |
| **R39.** Aplicar instrumento validado de avaliação da qualidade de vida para a população brasileira, preferencialmente a cada 12 meses, incluindo inventários para avaliação de dor crônica. | **Strong** | **100%** |
| **R40.** Solicitar exames subsequentes de ressonância magnética ou polissonografia apenas quando clinicamente indicado. | **Strong** | **100%** |
| **R41.** Avaliar aspectos relacionados à saúde mental de pacientes/famílias, especialmente sinais e sintomas de ansiedade e depressão, encaminhando para avaliação especializada quando necessário. | **Strong** | **100%** |
| ***Pharmacovigilance*** |  |  |
| **R42.** Monitorar efeitos adversos em todas as consultas; instruir os familiares a informar imediatamente eventos graves ao médico responsável. | **Strong** | **100%** |
| **R43.** Revisar medicamentos concomitantes a cada visita para identificar possíveis interações. | **Strong** | **100%** |
| ***Dose adjustment*** |  |  |
| **R44.** Avaliar a necessidade de ajuste de dose de vosoritida de acordo com a variação de peso, seguindo a recomendação de bula. | **Strong** | **100%** |
| **R45.** Reavaliar, a cada consulta, a resposta e as metas personalizadas de crescimento e ganhos funcionais. | **Strong** | **100%** |
| **Discontinuation of vosoritide therapy** |  |  |
| ***Criteria*** |  |  |
| **R46**. Na presença de velocidade de crescimento ≤1,5 cm por ano e/ou do fechamento completo das placas epifisárias (conforme verificado por medições repetidas e validadas para excluir erro de mensuração), suspender definitivamente a vosoritida. | **Strong** | **100%** |
| **R47**. A terapia também pode ser interrompida se o paciente atingir a estatura previamente definida como satisfatória para seus objetivos individuais. | **Strong** | **100%** |
| **R48**. Interromper o tratamento quando, após avaliação em múltiplas consultas e revisão de adesão, não houver ganho em termos de crescimento ou funcionalidade que justifique a continuidade, ou quando houver declínio funcional/dor inexplicável associado a outras condições subjacentes. | **Strong** | **100%** |
| **R49**. Suspender a vosoritida se o paciente ou a família não tolerarem as injeções diárias, mesmo após suporte técnico e psicológico adequados, ou se optarem voluntariamente pela interrupção, e oferecer aconselhamento sobre alternativas terapêuticas e suporte multidisciplinar. | **Strong** | **100%** |
| **R50**. Interromper temporariamente a medicação em situações que elevem o risco de hipotensão arterial (procedimentos cirúrgicos com restrição hídrica, vômitos, diarreia ou outras condições que reduzam ingestão ou aumentem perda de líquidos). | **Strong** | **100%** |
| **R51**. Na ocorrência de gestação, o tratamento deve ser interrompido pela ausência de dados que suportem a segurança do seu uso durante a gestação. | **Strong** | **100%** |
| **Post-vosoritide monitoring** |  |  |
| ***Guidance*** |  |  |
| **R52**. Orientar quanto a necessidade de manter acompanhamento clínico regular por toda a vida. | **Strong** | **100%** |
| **R53**. Planejar a transição do cuidado pediátrico para serviços de saúde especializados, se possível, do adulto de forma estruturada, iniciando orientações na adolescência e garantindo continuidade sem perda de seguimento. | **Strong** | **100%** |
| ***Assessment*** |  |  |
| **R54**. Avaliar, em cada consulta, de forma estruturada e multidisciplinar, as complicações frequentes da acondroplasia, encaminhando para avaliação especializada quando necessário. | **Strong** | **100%** |
| ***Education*** |  |  |
| **R55**. Disponibilizar recursos educativos a pacientes/famílias para favorecer a tomada de decisões informadas ao longo da vida adulta, reforçando o padrão de herança autossômica dominante da acondroplasia e suas implicações para planejamento familiar dentro de um contexto de aconselhamento genético. | **Strong** | **100%** |
