# Supplementary material for: Vosoritide therapy in Brazil: Consensus on the Diagnosis, Monitoring, and Management of Achondroplasia
Source: Orphanet J Rare Dis. 2026 Aug 6;21:396. doi: 10.1186/s13023-026-04519-8 (PMC13732916; doi:10.1186/s13023-026-04519-8)
Supplement: Supplementary file 2 [file 13023_2026_4519_MOESM2_ESM.docx]

**SUPPLEMENTARY MATERIAL**

Supplementary Table 2. Complementary Tests in Achondroplasia

| **Indication / Focus** | **Test / Assessment** | **Specific Recommendations** |
| --- | --- | --- |
| **Neurological** | | |
| **Foramen Magnum Stenosis** | **MRI** | Evaluate the cervicomedullary junction and foramen magnum size.  • **Target:** babies (consider during the first months of life).  • **Follow-up:** If complication is not present, close monitoring is required at least up to age 3 years. |
| **Spinal Alignment and Stenosis** | **MRI** | • **Baseline:** Recommended (where feasible) to monitor spinal alignment and stenosis.  • **Symptomatic:** Perform if symptoms occur (pain, tingling, cramping, muscle weakness, decline of walking distance, bowel/urinary incontinence, or spinal cord injury). |
| **Orthopedic** | | |
| **Lower Limb Angular Deformity** | **Radiograph** | • **Type:** Standing anterior–posterior radiographs.  • **Scope:** Full length of both legs (from hips to ankles).  • **Positioning:** With the patella facing forward. |
| **Lateral Knee Pain** | **MRI** | Should be considered to rule out the presence of a **discoid meniscus**. |
| **Airways** | | |
| **Upper Airway Obstruction and OSA** | **Polysomnography (Sleep Study)** | • **Type:** Overnight.  • **Target:** children (no later than 2 years of age) |
| **Hearing / Auditory** | **Audiological Assessment** | Should be performed by an otolaryngologist. |
| **Dental & Craniofacial** | **Preventative Dentistry Assessment** | Assessment of maxillary hypoplasia, relative mandibular prognathism, class III malocclusion, and macroglossia.  • **Taget:** children (5–6 years of age) |
| **Cardiology** | | |
| **Cardiovascular Health** | **Blood Pressure Monitoring** | Monitor regularly using a cuff that fits the arm appropriately.  • **Target:** adults |
| **Pediatric** | | |
| **Growth & Nutrition** | **Anthropometric Assessment** | • **Metrics:** Height, weight, and waist-to-hip ratio.  • **Records:** Dietary records should be monitored regularly. |
| **Body composition** | **Bioelectrical impedance analysis (BIA)** | Total body fat, percent body fat, and fat-free mass estimates. |

MRI: magnetic resonance imaging; OSA: obstructive sleep apnea.
